# *Plasmodium falciparum *gametocyte sex ratios in children with acute, symptomatic, uncomplicated infections treated with amodiaquine

**DOI:** 10.1186/1475-2875-7-169

**Published:** 2008-09-02

**Authors:** Akintunde Sowunmi, Sulayman T Balogun, Grace O Gbotosho, Christian T Happi

**Affiliations:** 1 Department of Pharmacology & Therapeutics and Institute for Medical Research and Training, University of Ibadan, Ibadan, Nigeria

## Abstract

**Background:**

Amodiaquine is frequently used as a partner drug in combination therapy or in some setting as monotherapy, but little is known about its effects on gametocyte production and sex ratio and its potential influence on transmission in Africa. The effects of amodiaquine on sexual stage parasites and gametocyte sex ratio, and the factors associated with a male-biased sex ratio were evaluated in 612 children with uncomplicated *Plasmodium falciparum *malaria who were treated with amodiaquine during the period 2000 – 2006 in an endemic area.

**Methods:**

Clinical, parasitological and laboratory parameters were evaluated before treatment and during follow-up for 28–42 days, and according to standard methods. Gametocyte sex ratio was defined as the proportion of peripheral gametocytes that are male.

**Results:**

Clinical recovery from illness occurred in all children. Gametocytaemia was detected in 66 patients (11%) before treatment and in another 56 patients (9%) after treatment. Gametocyte densities were significantly higher by days 3–7 following treatment compared with pre-treatment (P < 0.0001). Overall, mean gametocyte sex ratio increased significantly during follow-up and over the study periods from 2000–2006 (P < 0.001 in both cases), but was female-biased at enrolment throughout the study periods. Absence of fever, a haematocrit < 25%, asexual parasitaemia > 20,000/μL, gametocytaemia < 18/μL, and enrolment in 2006 were associated with a male-biased sex ratio pre-treatment. Anaemia and high parasitaemia were independent predictors of gametocyte maleness 7 days post treatment.

**Conclusion:**

Amodiaquine may significantly increase gametocyte carriage, density and sex ratio, and may potentially influence transmission. It is possible that anaemia could have contributed to the increased sex ratio. These findings may have implications for malaria control efforts in Africa.

## Background

Amodiaquine, a Manic base related to chloroquine, is considered a safe drug for the treatment of acute, uncomplicated, *Plasmodium falciparum *malaria [1], and is increasingly used as partner drug for artemisinin [2-7] and non-artemisinin based [8-10] combination therapies. Studies have shown that antimalarials modify gametocyte carriage and influence malaria transmission [7,11-14], suggesting careful consideration in the selection of partner drugs in combination therapies.

Despite similarity and superior efficacy to chloroquine, increasing use in Africa, and the suggestion that mutations conferring resistance to chloroquine may confer resistance to amodiaquine in Africa [15,16], little is known of the effects of amodiaquine on gametocyte carriage and sex ratio, and its potential influence on transmission in African children. Such a study is necessary as it may potentially harness the efforts to control drug resistance and prolong the use of combination therapies in the community.

The aims of the present study were to: determine the effects of amodiaquine on asexual-stage parasites, gametocyte carriage and sex ratio; and evaluate the factors that influence the production of a male-biased sex ratio in children presenting with acute, symptomatic, uncomplicated *P. falciparum *malaria before and following treatment with amodiaquine in an endemic area.

## Patients and methods

### Patients

The study was conducted in children aged ≤ 12 years with acute, uncomplicated, *P. falciparum *malaria in Ibadan, a malaria endemic area in southwestern Nigeria [17]. Fully informed consent was obtained from the parents/guardians of each child. Inclusion criteria were: fever or history of fever in the 24–48 h preceding presentation, pure *P. falciparum *parasitaemia ≥ 2,000 asexual forms/μL, absence of other concomitant illness, no history of antimalarial use in the 2 weeks preceding presentation, and negative urine tests for antimalarial drugs (Dill-Glazko and lignin). Patients with severe malaria [18], severe malnutrition, serious underlying diseases (renal, cardiac, or hepatic), and known allergy to study drug were excluded from the study. The study was approved by the Ethics Committee of the Ministry of Health, Ibadan, Nigeria.

### Drug management

After clinical assessment, blood was obtained for haematocrit determination and for quantification of asexual and sexual parasitaemia. Patients were treated with 25–30 mg/kg of amodiaquine base (Camoquine^®^) given orally over 3 days. All patients waited for at least 3 h after to ensure the drug was not vomited. If it was, the patient was excluded form the study.

Oral paracetamol (acetaminophen) at 10–15 mg/kg 6–8 hourly was given for 12–24 h if body temperature was > 38°C. Patients were seen daily, at approximately the same time of the day for the first four days (days 0–3) and then daily on days 7, 14, 21, 28, 35 and 42 after treatment had begun. At each visit, patients were assessed clinically, and thick and thin blood smears were obtained for quantification of parasitaemia. The fever clearance time (FCT) was defined as the time taken for the body temperature to fall below 37.5°C and remain below this value for > 48 h.

### Laboratory investigations

Asexual parasite and gametocyte counts were measured daily for the first four days (days 0–3) and thereafter on days 7, 14, 21, 28, 35 and 42. Quantification in Giemsa-stained thick blood films was undertaken against 500 leukocytes in the case of asexual parasitaemia, and against 1000 leukocytes in the case of gametocytes, and from this figure, the parasite density was calculated assuming a leukocyte count of 6,000/μL of blood. Parasite clearance time (PCT) was the time interval from the start of antimalarial treatment until the asexual parasite count fell below the detectable levels in a peripheral blood smear. Capillary blood, collected before and during follow-up, was used to measure packed cell volume (PCV). PCVs were measured using a microhaematocrit tube and microcentrifuge (Hawksley, Lancing, UK). Routine haematocrit was undertaken on days 0, 3, 7, 14, 21, 28, 35 and 42. Blood was spotted on filter papers on days 0, 1, 3, 7, 14, 21, 28, 35 and 42 and at the time of re-appearance of peripheral parasitaemia after its initial clearance for parasite genotyping. Molecular genotyping was carried out as previously described [16].

### Determination of gametocyte sex ratio

Gametocyte sex determination was based on the following criteria [19,20]: males (microgametocytes) are smaller than females (macrogametocytes), the nucleus is larger in males than females, the ends of the cells are rounder in males and angular in females, with Giemsa the cytoplasm stains purple in males and deep blue in females, and the granules of malaria pigment are centrally located females and more widely scattered in males. The sex ratio was defined as the proportion of gametocytes in peripheral blood that were male [21]. Gametocytes were sexed if the gametocyte density was ≥ 10/μL blood.

### Data analysis

Data were analyzed using version 6 of the Epi-Info software [22] and the statistical programme SPSS for Windows version 10.01 [23]. Variables considered in the analysis were related to the densities of *P. falciparum *gametocytes and trophozoites. Proportions were compared by calculating χ^2 ^with Yates' correction or by Fisher exact or by Mantel Haenszel tests. Normally distributed, continuous data were compared by Student's t-tests and analysis of variance (ANOVA). Data not conforming to a normal distribution were compared by the Mann-Whitney U-tests and the Kruskal-Wallis tests (or by Wilcoxon ranked sum test). Kaplan-Meier plots are also presented to compare gametocyte carriage rates, and the duration of carriage of a male biased gametocyte sex ratio following treatment in those who were gametocytaemic at presentation. Differences in survival time were assessed by inspection of Kaplan-Meier curves and log-rank tests. The relationship between gametocyte sex ratio and gametocyte or asexual parasite density was assessed by linear regression. A multiple logistic regression model was used to test the association between a male biased sex ratio, that is, sex ratio ≥ 0.5 (yes or no at presentation or during follow up) and factors that were significant at univariate analysis: presence of fever, haematocrit < 25%, asexual parasitaemia > 20,000/μL, and gametocytaemia < 18/μL. Because the study was conducted over a period of six and a half year, time in years since the commencement of the study was included as a covariate in the model for pre-treatment male-biased sex ratio. All tests of significance were two-tailed. P-values of ≤ 0.05 were taken to indicate significant differences. Data were (double)-entered serially using the patients' codes and were only analyzed at the end of the study.

## Results

### Baseline characteristics of patients at enrolment

Between September 2000 and December 2006, 615 children (297 males, 318 females) with *P. falciparum *malaria, aged between 0.5–12 years (mean ± standard deviation [SD] = 6.5 ± 3.2 years) were enrolled. Of these, 612 (294 males, 318 females) completed at least 28 days of follow up and were analyzed (Table 1). The characteristics of the children were similar during the study periods except for the geometric mean parasite density that was significantly higher in 2006 than in 2000 and 2004 (P < 0.0001) (Table 1).

**Table 1:** Overall baseline characteristics and immediate therapeutic response of the children enrolled into the study, and by year of enrolment

		Year			
		
		2000	2004	2006	2000–2006
Enrolled	N	105	390	120	615
Gender	F/M	53/52	195/195	70/50	318/297
Age (years)	Mean	5.6	6.7	6.7	6.5
	SD	2.7	3.3	3.2	3.2
Weight (kg)	Mean	15.6	18.1	18.6	17.8
	SD	4.9	7.1	6.8	6.8
Packed cell volume (%)	Mean	29.8	30	31.2	30.3
	SD	3.9	3.9	3.9	3.9
Duration of illness (days)	Mean	2.9	2.9	2.9	2.9
	SD	1.2	1.2	1.1	1.2
Temperature (°C)	Mean	38.2	38.2	38.1	38.1
	SD	1.2	1.2	1.2	1.2
Asexual parasitaemia (/μL)	GM	21,261	21,468	68,839*	26,908
Gametocytaemia (/μL)	GM	13	15	17	15
Parasite clearance time (days)	Mean	2.6	2.6	2.8	2.7
	SD	0.8	0.7	1.7	1.3
Fever clearance time (days)	Mean	1.1	1.1	1.1	1.1
	SD	0.4	0.4	0.3	0.4

*P = < 0.0001

SD, standard deviation; GM, geometric mean.

### Clinical responses

All children responded promptly to treatment, and none developed severe malaria. The overall mean (range) FCT was 1.1 (SEM 0.02) d, and was not significantly different between the years of enrolment (Table 1). None of the studied children had significant adverse effects as monitored by clinical symptoms, but overall, 46 children reported pruritus, which did not interfere with sleep.

### Parasitological responses

The overall mean PCT (SEM) was 2.7 (0.005) d and was not significantly different during the study periods (Table 1). Recrudescent infections confirmed by polymerase chain reaction (PCR) occurred in 25 children: in 6, 8, and 11 children in 2000, 2004, and 2006, respectively. The characteristics of the recrudescent infections will be reported elsewhere.

### Gametocytaemia

Gametocytes were detected in peripheral blood in 122 (20%) children (in 66 children before treatment and in 56 children after initiation of treatment) (Table 2). Gametocyte detection rates before, during and after treatment were similar during the study periods (Table 3). Gametocyte densities during the study periods are summarized in Table 3. Overall, gametocytaemia increased significantly by days 3–7 during follow up (P < 0.0001) but did not differ between the study periods.

**Table 2:** Gametocyte carriage before and after treatment in 612 children who completed 28-days follow up

	Gametocyte carriage				
		
Year	No.	At enrolment	After treatment	Total	P value
2000	104	12 (11.5)	13 (12.5)	25	1.0
2004	388	38 (9.8)	29 (7.5)	67	0.31
2006	120	16 (13.3)	14 (11.7)	30	0.85
2000–2006	612	66 (10.8)	56 (9.2)	122	0.39

**Table 3:** Gametocyte density before and after amodiaquine treatment of the 612 children who completed 28-day follow-up

		Year				
			
Days of follow up	Gametocyte density (/μL)	2000	2004	2006	2000–2006	P value
0	GM	13 (12)	16 (38)	17 (16)	16 (66)	0.66
	Range	6 – 498	6 – 288	6 – 138	6 – 498	
1	GM	13 (4)	17 (37)	24 (8)	18 (49)	0.85
	Range	6 – 48	6 – 648	6 – 114	6 – 648	
2	GM	21 (7)	20 (34)	35 (6)	21 (47)	0.77
	Range	6 – 174	6 – 600	6 – 396	6 – 600	
3	GM	18 (11)	25 (38)	19 (21)	23 (70)	0.75
	Range	6 – 210	6 – 720	6 – 798	6 – 798	
7	GM	10 (12)	32 (22)	22 (16)	22 (50)	0.3
	Range	6 – 24	6 – 600	6 – 558	6 – 600	
14	GM	11 (5)	14 (16)	11 (8)	13 (29)	0.7
	Range	6 – 18	6 – 108	6 – 180	6 – 180	
21	GM	-	9 (4)	18 (1)	10 (5)	0.36
	Range		6 – 12	18	6 – 18	
28	GM	-	12 (2)	57 (2)	26 (4)	0.82
	Range		12	6 – 546	6 – 546	

GM, geometric mean; Number in parenthesis indicates number of patients

### Duration of gametocyte carriage in children with gametocytaemia at enrolment

**Figure 1:**
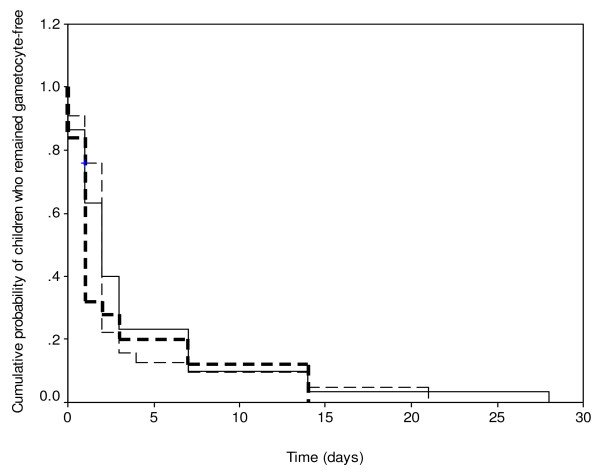
Kaplan-Meier plot (survival curve) of cumulative probability of remaining gametocyte-free after amodiaquine treatment of malarious children in 2000 (thick broken line), 2004 (thin broken line) and 2006 (solid line) (Log rank statistic = 0.8, P = 0.23).

The probability of a mosquito infectivity following a blood meal is related to gametocyte density and the duration of carriage by the host. Figure 1 is a Kaplan-Meier plot (survival curve) of the cumulative probability of remaining gametocyte free following treatment with amodiaquine during the study periods. The probabilities were similar during the periods of the study (Log rank statistic = 0.8, P = 0.23).

### Temporal changes in gametocyte sex ratio

In 122 children who were gametocytaemic at presentation or during follow up, a total of 2,286, 2,197, 3,108, 4,676, 2,880, 618, 54 and 576 gametocytes were counted on days 0, 1, 2, 3, 7, 14, 21, and 28, respectively. Of these, 2,050, 1,986, 2,843, 4,518, 2,699, 600, 54, and 440 gametocytes could be sexed on days 0, 1, 2, 3, 7, 14, 21, and 28, respectively. The corresponding number of patients in whom the gametocytes were counted was 66, 49, 47, 70, 50, 29, 5, and 4, respectively.

**Figure 2:**
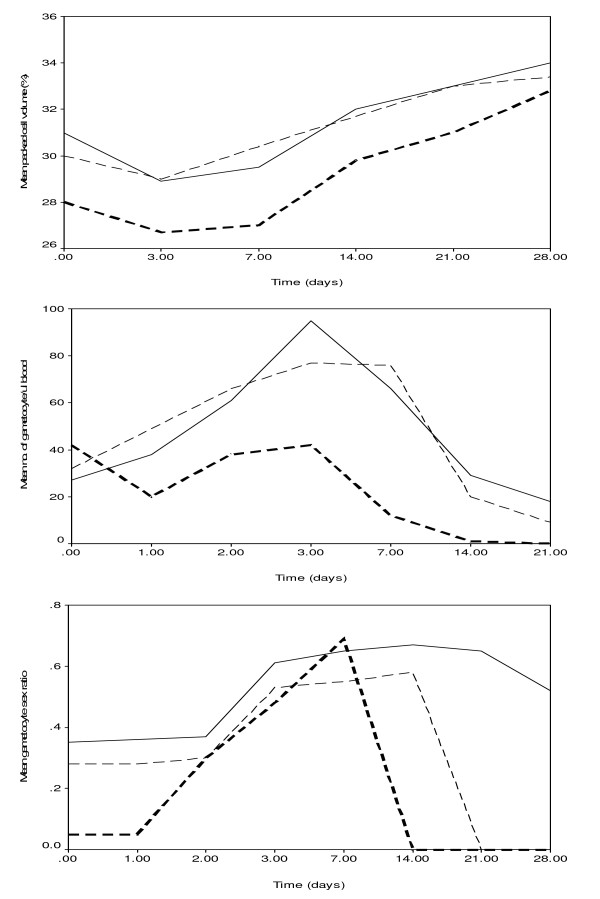
Variations in the packed cell volume, density of gametocyte, and gametocyte sex ratio over the course of treatment of malaria infections with amodiaquine in years 2000 (thick broken line), 2004 (thin broken line), and 2006 (solid line).

Following treatment with amodiaquine, gametocyte sex ratio increased significantly over the course of the infection. (Figure 2 and Table 4): 7.6% of the gametocytes were male at day 0, 25% at day 3, and 29% at day 7 (X^2 ^= 15.2, P = 0.0005). Gametocyte sex ratio at enrolment increased significantly during the study periods: the ratio was 0.4% in 2000, 4.8% in 2004, and 20% in 2006 (P < 0.0001), although it was still largely female-biased (Table 4). The variations in haematocrit, density of gametocyte and gametocyte sex ratio are shown in Figure 2. During the first week of follow up, gametocyte sex ratio increased as the packed cell volume decreased and the gametocyte density increased, suggesting that the latter may have been a direct contribution of the effect of amodiaquine on sex ratio, since, in general, low but not relatively high gametocytaemia is associated with male-biased sex ratio.

**Table 4:** Variations in mean gametocyte sex ratio with time after amodiaquine treatment of 612 children

		Year				
			
Days of follow up	Sex ratio	2000	2004	2006	2000–2006	P value
0	Mean	0.004 (12)	0.048 (38)	0.2 (16)	0.076 (66)	< 0.0001
	SE	0.004	0.019	0.05	0.019	
1	Mean	0.0 (4)	0.076 (37)	0.32 (8)	0.11 (49)	0.003
	SE	0.0	0.025	0.12	0.03	
2	Mean	0.13 (7)	0.1 (34)	0.25 (6)	0.13 (47)	0.3
	SE	0.08	0.03	0.08	0.03	
3	Mean	0.29 (11)	0.15 (38)	0.4 (21)	0.25 (70)	0.006
	SE	0.1	0.03	0.08	0.04	
7	Mean	0.25 (12)	0.25 (22)	0.37 (16)	0.29 (50)	0.48
	SE	0.11	0.05	0.1	0.05	
14	Mean	0.13 (5)	0.28 (18)	0.51 (6)	0.3 (29)	0.14
	SE	0.08	0.08	0.14	0.06	
21	Mean	-	0.25 (4)	0.3 (1)	0.26 (5)	-
	SE	-	0.25	-	0.19	
28	Mean	-	0.0 (2)	0.52 (2)	0.26 (4)	0.46
	SE	-	0.0	0.19	0.17	

SE, standard error of mean; Number in parenthesis indicates number of patients

**Figure 3:**
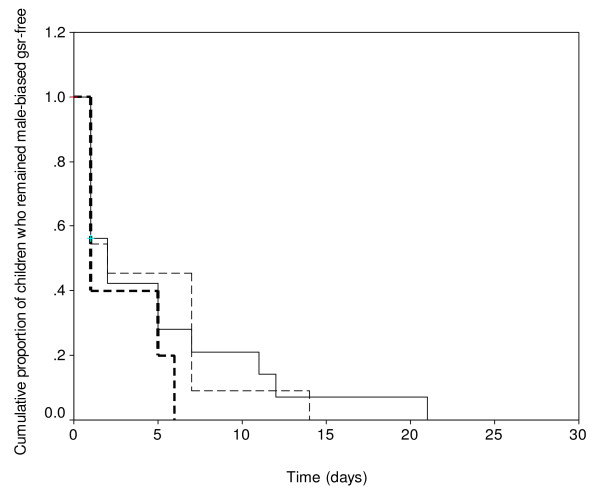
Kaplan-Meier plot (survival curve) of cumulative probability of remaining free of a male-biased gametocyte sex ratio (gsr) after amodiaquine treatment of malarious children in 2000 (thick broken line), 2004 (thin broken line) and 2006 (solid line) (Log rank statistic = 2, P = 0.37).

The possibility that gametocyte infectivity may be related to the duration of carriage of a less female biased sex ratio was examined. Figure 3 is a Kaplan-Meier plot of the cumulative probability of remaining a less female-biased sex ratio free following treatment with amodiaquine during the study periods. The probabilities were similar during the periods of the study (Log rank statistic = 2, P = 0.37).

### Relationship between gametocyte sex ratio and gametocytaemia or asexual parasitaemia

The percentage of male gametocytes was negatively correlated with gametocyte density but not with asexual parasite density (Spearman's r = -0.46, P < 0.0001 and r = 0.009, P = 0.94, respectively with n = 66 in both cases.

### Risk factors associated with a male-biased gametocyte sex ratio

The pre-treatment factors associated with a male-biased sex ratio are shown in Table 5. Age, gender or duration of illness before presentation appeared not to be associated with a male-biased sex ratio at enrolment. Absence of fever, a haematocrit < 25%, asexual parasitaemia > 20,000/μL, gametocytaemia < 18/μL, and enrolment in 2006 were significantly associated with a male-biased sex ratio at enrolment.

**Table 5:** Risk factors for a male-biased gametocyte sex ratio at presentation in children with uncomplicated falciparum malaria, showing the crude odd ratios (OR) and their 95% confidence intervals (CI)

Variables	Total Number	No with gsr ≥ 0.5	OR (95% CI)	P value
Gender				
male	37	2	1	
female	29	3	2 (0.4 – 9.8)	0.45
				
Age				
< 5 years	29	1	1	
> 5 years	37	4	3.4 (0.36 – 32)	0.26
				
Duration of illness				
≤ 3 days	54	5	1	
≥ 3 days	12	0	0.9 (0.8 – 1)	0.16
				
Fever*				
afebrile	29	5	1	
febrile	36	0	0.8 (0.7 – 1)	0.01
				
Packed cell volume				
≤ 25%	11	3	1	
≥ 25%	43	2	0.1 (0.02 – 0.9)	0.02
				
Parasitaemia				
≤ 20,000 μl/l	33	0	1	
> 20,000 μl/l	33	5	1.2 (1 – 1.4)	0.02
				
Gametocytaemia				
< 18 μl/l	38	5	1	
≥ 18 μl/l	28	0	0.8 (0.7 – 1)	0.04
				
Year				
Pre 2006	50	1	1	
2006 onwards	16	4	16 (1.7 – 159.7)	0.002

*Fever, body temperature ≥ 37.5°C

Following treatment, a haematocrit < 25% on days 0 and 7 and a parasitaemia greater > 20,000/μL of blood were independent predictors of a male biased sex ratio 7 days post initiation of treatment with amodiaquine (Table 6).

**Table 6:** Risk factors for a male-biased gametocyte sex ratio 7 days after amodiaquine treatment of children with uncomplicated falciparum malaria, showing the crude and adjusted odd ratios (OR) and their 95% confidence intervals (CI)

Variables	Total Number	No with gsr ≥ 0.5	OR (95% CI)	P value	AOR (95% CI)	P value
Gender						
male	25	7	1			
female	25	8	1.1 (0.6 – 2)	0.75	-	-
						
Age						
< 5 years	20	7	1			
≥ 5 years	30	8	0.8 (0.5 – 1.4)	0.5	-	-
						
Duration of illness						
≤ 3 days	38	12	1			
> 3 days	12	3	0.8 (0.2 – 2.5)	0.67	-	-
						
Fever*						
afebrile	21	6	1			
febrile	29	9	1.1 (0.6 – 1.7)	0.85	-	-
						
PCV (day 0)						
< 25%	6	5	1		1	
≥ 25%	44	10	0.6 (0.5 – 1)	0.002	0.06 (0.006 – 0.7)	0.03
						
Parasitaemia (day 0)						
≤ 20,000 μl/l	22	3	1		1	
> 20,000 μl/l	28	12	1.8 (1.1 – 2.7)	0.025	5.7 (1 – 33)	0.05
						
Gametocytaemia (day 0)						
< 18 μl/l	11	4	1			
≥ 18 μl/l	17	7	1.1 (0.6 – 2)	0.8	-	-
						
Year						
pre 2006	34	8	1			
2006	16	7	1.8 (0.8 – 4)	0.15	-	-
						
GSR (day 0)						
< 0.5	26	10	1			
≥ 0.5	2	1	1.5 (0.1 – 22)	0.75	-	-
						
FCT						
< 2 days	33	10	1			
≥ 2 days	2	1	2.2 (0.1 – 32)	0.56	-	-
						
PCT						
< 3 days	32	8	1			
≥ 3 days	18	7	1.5 (0.7 – 3.1)	0.3	-	-
						
PCV (day 7)						
< 25%	4	3	1		1	
≥ 25%	46	12	0.8 (0.6 – 3.7)	0.04	0.06 (0.003 – 1)	0.05
						
Re-parasitaemia						
Absent	44	14	1			
Present	6	1	0.5 (0.06 – 3.7)	0.45	-	-

*Fever, body temperature ≥ 37.5°C; FCT, fever clearance time; GSR, gsr, gametocyte sex ratio; PCV, packed cell volume; PCT, parasite clearance time

## Discussion

The study showed continuing efficacy of amodiaquine against asexual parasites, relative lack of gametocytocidal effects, and peak gametocyte carriage occurring 3–7 days after treatment commenced. The last finding is in contradistinction to that following treatment with artemisinin derivatives or artemisinin-based combination therapy where peak gametocyte carriage is seen pre-treatment in children from this endemic area [7,10,24].

The average sex ratio of 0.076, is considerably lower than those reported from Senegal (0.346) [25] or Cameroon (0.22) [20]. Sex ratio increased over the relatively long period but the causes are unclear. Exposure of these children to sex ratio modifying influences, including antimalarial drugs [13,26] may have been contributory. Sex ratio may be positively correlated with gametocyte density in animal infections [21,27] but in the present study was significantly negatively correlated with gametocytaemia – a finding similar to that from Senegal [25].

Although significant variations in sex ratio may occur in natural populations [28,29], a consistent finding during the entire period of the study was a significant shift in sex ratio towards maleness by day 7 of initiation of treatment. Indeed by this time, 29% of gametocytes were male. It would appear anaemia was an important contributor to gametocyte maleness and had resolved in many children by day 7 (Figure 3). However, overall, the absence of low gametocytaemia, which was expected to enhance gametocyte maleness as a form of fertility insurance [30], and the significantly increased gametocytaemia on day 7 (a possible effect of amodiaquine) suggest that by some as yet unknown way, amodiaquine may encourage the development of a less female-biased gametocyte sex ratio. A possible explanation is that amodiaquine or its metabolite could exert different effects on male and female gametocytes particularly if the gametocytes are exposed to the metabolite for a long time. Perhaps by an effect on sex-specific half-lives as has been previously reported for pyrimethamine-sulfadoxine [13] but it is yet to be investigated. Alternatively sequestered gametocytes with a less female biased sex ratio could have been released during this period. An interesting finding was the year to year variation in sex ratio. The reasons for this are unclear but may not be unrelated to environmental and other cues resulting in adaptive changes by the parasites.

Robert and others [25] found that in a population of symptomatic and asymptomatic individuals, anaemia and a wave of gametocytes were associated with significant effects on sex ratio. In the present data set, five factors were associated with increased risk of a less female biased sex ratio at enrolment: absence of fever, anaemia, asexual parasitaemia > 20,000/μL, low gametocytaemia and enrolment in the year 2006. The differences in the risk factors between the present study and that of Robert *et al *[25] clearly are due to differences in design. However, that anaemia is associated with a less female biased sex ratio may work in concert with other factors to promote the male biased sex ratio often observed following chemotherapy of the disease.

In lizard malaria parasite infections, high levels of parasitaemia are associated with a less female-biased sex ratio [21]. In the cohorts of children, high levels of parasitaemia, which are common in young children in this endemic area, were also found to be associated with a male-biased sex ratio. This was not unexpected – high levels of parasitaemia are associated with low gametocytaemia [14] – a relatively reduced risk of gametocyte carriage and a factor that promotes gametocyte maleness as a form of fertility insurance [30]. In addition, in areas of intense transmission, such as ours, a less female biased sex ratio is favoured [31] primarily because of multiplicity of infections, on the average the number of infecting clones in the area is about 3–4 [32,33].

Absence of fever is associated with increased risk of gametocyte carriage [12,14,34]. It is unclear why afebrile children should have an increased risk of male biased sex ratio, and we have no explanation for our finding. Anaemia before, during, and after treatment was an important predictor of a male biased sex ratio following treatment with amodiaquine. This was expected and it supports the suggestion that this factor may contribute to antimalarial-associated gametocyte maleness. If artemisinin derivatives, as has been proposed, selectively favour the emergence of a female-biased sex ratio [7], it would now be important to evaluate the predictors of male biased sex ratio in children treated with different antimalarial drugs.

There is a need to justify the sex ratio regarded as male biased in our study. Traditionally, sex ratio is assigned by microscopy that often overestimated the proportion of female gametocytes [35]. Therefore, regarding a sex ratio ≥ 0.5 as male biased clearly shows a significantly male biased ratio. Sex ratio can now be more accurately estimated by a recently developed reverse-transcription polymerase chain reaction techniques [36].

A limitation of the present study is that only gametocytaemia ≥ 10/μl were sexed. A limitation on the predictors of less female biased sex ratio is the inability to evaluate other potential predictors such as treatment with other gametocyte sex ratio modifying drugs such as pyrimethamine-sulphadoxine, trimethoprim-sulphamethoxazole [13,25]. This will be examined in an ongoing study. However, the present results are strengthened by the use of a consistent protocol over the entire study period; the 6.5 year-long period allowed the factoring of time into the analysis and to gain some insight into the evolution of a less female biased gametocyte sex ratio in our area of study, in a manner similar to our observations on the evolution of drug resistance in our study area [37].

## Conclusion

Amodiaquine may significantly increase gametocyte carriage, density and sex ratio in African children treated for falciparum malaria, and may potentially influence transmission. It is also possible that anaemia could have contributed to the less female biased sex ratio.

## Conflict of interests

The authors declare that they have no competing interests.

## Authors' contributions

AS led the design, conduct, data analysis and manuscript preparation. STB was involved in data analysis and manuscript preparation. GOG and CTH were involved in the design, conduct, and preparation of the manuscript. All authors read and approved the final draft of the manuscript.
